# Adaptive VMD–K-SVD-Based Rolling Bearing Fault Signal Enhancement Study

**DOI:** 10.3390/s23208629

**Published:** 2023-10-22

**Authors:** Meijiao Mao, Kaixin Zeng, Zhifei Tan, Zhi Zeng, Zihua Hu, Xiaogao Chen, Changjiang Qin

**Affiliations:** 1 School of Mechanical Engineering and Mechanics, Xiangtan University, Xiangtan 411105, China; maomj2000@xtu.edu.cn (M.M.); 202121542052@smail.xtu.edu.cn (K.Z.); 202121542054@smail.xtu.edu.cn (Z.Z.); 202221542158@smail.xtu.edu.cn (Z.H.); chenxiaogao@xtu.edu.cn (X.C.); qincj@xtu.edu.cn (C.Q.); 2 Engineering Research Center of Complex Trajectory Machining Process and Equipment, Ministry of Education, Xiangtan University, Xiangtan 411105, China

**Keywords:** rolling bearing, arithmetic optimization algorithm, variational mode decomposition, K-singular value decomposition

## Abstract

To address the challenges associated with nonlinearity, non-stationarity, susceptibility to redundant noise interference, and the difficulty in extracting fault feature signals from rolling bearing signals, this study introduces a novel combined approach. The proposed method utilizes the variational mode decomposition (VMD) and K-singular value decomposition (K-SVD) algorithms to effectively denoise and enhance the collected rolling bearing signals. Initially, the VMD method is employed to separate the overall noise into intrinsic mode functions (IMFs), reducing the noise content within each IMF. To optimize the mode component, K, and the penalty factor, α, in VMD, an improved arithmetic optimization algorithm (IAOA) is employed. This ensures the selection of optimal parameters and the decomposition of the signal into a set of IMFs, forming the original dictionary. Subsequently, the signals are decomposed into multiple IMFs using VMD, and an original dictionary is constructed based on these IMFs. K-SVD is then applied to the original dictionary to further reduce the noise in each IMF, resulting in a denoised and enhanced signal. To validate the efficacy of the proposed method, rolling bearing signals collected from Case Western Reserve University (CWRU) and thrust bearing test rigs were utilized. The experimental results demonstrate the feasibility and effectiveness of the proposed approach in denoising and enhancing the rolling bearing signals.

## 1. Introduction

Rolling bearings play a crucial role as key support elements in large-scale industrial production. The health and proper functioning of these bearings directly impact the normal operation of specific organizations [1,2,3]. Consequently, assessing the operational reliability of rolling bearings, monitoring their operating status, and conducting fault diagnosis are essential for ensuring the safe and stable operation of machinery and equipment. By implementing predictive maintenance strategies for rolling bearings, productivity can be improved, and the likelihood of production accidents can be reduced [4,5,6,7,8]. To achieve this, it is necessary to conduct comprehensive assessments of rolling bearing health, monitor their performance, and diagnose faults promptly.

Signal-based time–frequency domain analysis is widely employed as a means of diagnosing faults in rolling bearings. The signals generated during mechanical operation encompass a wealth of information pertaining to equipment performance; through signal analysis, it becomes possible to discern the operational state of rolling bearings [1,9]. Among the various types of signals used in rolling bearing analysis, such as vibration signals, acoustic signals, acceleration signals, and speed signals, vibration and acoustic signals are particularly advantageous due to their ability to capture subtle and higher-scale information. Hence, time–frequency domain analysis based on vibration and acoustic signals offers enhanced accuracy and sensitivity [10,11,12]. However, the vibration and acoustic signals collected often contain a considerable amount of redundant noise. Therefore, it is crucial to minimize the noise present in the raw acoustic signals and amplify the fault characteristic signals to ensure accurate fault diagnosis and analysis.

Variational mode decomposition (VMD), introduced by Dragomiretskiy and Zosso in 2014 [13], is a novel signal decomposition method that offers comparable performance to other methods such as empirical mode decomposition (EMD) [14], complete ensemble empirical mode decomposition (CEEMD) [15], and complete ensemble empirical mode decomposition with adaptive noise (CEEMDAN) [16]. VMD stands out due to its enhanced stability, robustness, and the avoidance of mode aliasing. Although the effectiveness of VMD is influenced by the mode component, K, and the penalty factor, α, optimizing these parameters using heuristic intelligent optimization algorithms has been recognized as an effective approach [17]. Numerous researchers have employed various intelligent optimization algorithms to fine-tune the parameters of VMD and analyze the resulting IMF to assess the operational status of bearings. Zhang et al. [18] investigated the mode component, K, and penalty factor, α, of VMD using correlation coefficients and energy ratios. They demonstrated the signal decomposition capability of VMD by decomposing both simulated and actual vibration signals under appropriate parameter settings. Zhang et al. [19] utilized the grasshopper optimization algorithm (GOA) to optimize the parameters in VMD. According to the research conducted by the aforementioned scholars, VMD has been identified as a viable signal processing method for decomposing bearing signals. However, the effectiveness of VMD’s decomposition is influenced by certain parameters. To enhance the decomposition performance to its fullest potential, this paper proposes the utilization of the IAOA to optimize the relevant parameters of VMD. The primary objective is to achieve a more comprehensive decomposition of bearing signals by fine-tuning these parameters.

K-singular value decomposition (K-SVD) is an algorithm originally proposed by M. Aharon [20] for designing an ultra-complete dictionary suitable for sparse representation. In comparison with other algorithms for signal sparse representation, K-SVD employs an iterative process of sparse coding and dictionary atom updating based on the current set of example signals. This iterative process leads to a better fit to the data; the dictionary columns are updated with the sparse representations, resulting in accelerated convergence. One of the advantages of K-SVD is its adaptability, as it can be combined with other tracking methods. Lebrun et al. [21] utilized K-SVD for image denoising and achieved favorable results, demonstrating the algorithm’s stability and noise reduction capabilities. Anaraki et al. [22] focused on dictionary construction in K-SVD and utilized compressed sensing measurements based on signals for sparse representation, showcasing the algorithm’s proficiency in noise reduction. Wu et al. [23] constructed dictionaries using different fabric samples and successfully reconstructed the samples using K-SVD, thus confirming the effectiveness of the algorithm. Wang et al. [24] proposed a method that combines the orthogonal matching pursuit (OMP) algorithm with K-SVD, illustrating the feasibility of K-SVD for noise reduction in signals from rotating machinery, such as rolling bearings. Based on the research conducted by the aforementioned scholars, K-SVD has been recognized as an effective method for noise reduction and signal decomposition. The decomposition effect of K-SVD is influenced by the choice of the original dictionary. To optimize the decomposition effect and enhance its performance, this paper proposes a novel approach. It utilizes the matrix composed of IMFs obtained through VMD as the original dictionary for K-SVD. By employing the IMFs from VMD as the initial dictionary, the aim is to improve the noise reduction capability of K-SVD to its maximum potential.

In conclusion, K-SVD is a powerful algorithm for sparse representation and has demonstrated good stability and noise reduction capabilities in various applications, including signal and image denoising. Its effectiveness is often limited by the constraints of the initially constructed dictionary. On the other hand, VMD is a novel signal processing method that offers advantages in signal decomposition and noise reduction. It overcomes issues such as endpoint effects and mode aliasing, resulting in its wide use in signal processing. However, the decomposition performance of VMD is influenced by the mode component, K, and the penalty factor, α. In this paper, an approach is proposed to optimize the relevant parameters of VMD using the IAOA. The IMFs obtained from VMD are then used to construct the original dictionary in K-SVD. This approach ensures the optimal performance of VMD while leveraging the noise reduction and enhancement capabilities of K-SVD. The proposed method was validated using experimental signals from rolling bearings obtained from Case Western Reserve University and Thrust Bearing Test Bed.

## 2. VMD–K-SVD-Based Signal Enhancement Method for Fault Characterization

### 2.1. VMD Basic Principle

The operation of the VMD algorithm can be understood as a variational process aimed at decomposing the original signal into IMFs. Each IMF represents a bandwidth-limited AM (amplitude modulation) and FM (frequency modulation) function. The expression for an IMF can be written as follows:

(1) $$\mu_{k}\left(t\right)=A_{k}\left(t\right)\cos\left(\varphi_{k}\left(t\right)\right)$$

where $A_{k}(t)$ is the instantaneous amplitude of $U_{k}(t)$ and $\phi_{k}(t)$ is the phase.

The VMD algorithm involves the construction and solution of variational problems. The construction of variational problems in VMD can be described as follows:

(1) The IMF components are subjected to a Hilbert transform, which introduces a unit pulse signal, δ(t). This process allows us to obtain the expression of the analyzed signal with K IMF components. The expression for the analyzed signal with K IMF components can be represented using Equation (2):
  (2) $$F_{k}=\left(\delta_{t}+\frac{j}{\pi t}\right)\mu_{k}\left(t\right)$$
(2) The resolved signal of the K IMF components is shifted to the baseband, and the center frequency of each IMF is adjusted using an exponential term, $e^{-jw_{k}t}$. This process results in an expression for the shifted spectrum, which can be represented as shown in Equation (3):
  (3) $$H_{k}=\left[\left(\delta_{t}+\frac{j}{\pi t}\right)\mu_{k}\left(t\right)\right]e^{-j\omega_{k}t}$$
  where $\omega_{k}(t)$ is the center frequency of each IMF component.
(3) The bandwidth of the signal is estimated using Gaussian smoothness, which involves calculating the square of the two-parameter gradient. This estimation helps in obtaining the expression for the constrained variational model. The constrained variational model expression can be represented as shown in Equation (4):
  (4) $$\{\begin{array}{c} \underset{\left\{\mu_{k}\right\},\;\left\{\omega_{k}\right\}}{\min}\left\{\sum_{k=1}^{K}\|{\partial_{t}\left[\left(\delta \left(t\right)+\frac{j}{\pi t}\right)\times \mu_{k}\left(t\right)\right]e^{-j\omega_{k}t}\|}_{2}^{2}\right\}\\ s.t.\sum_{k=1}^{K}\mu_{k}\left(t\right)=f\left(t\right) \end{array}$$

In the construction of variational problems in VMD, a penalty factor is introduced, and an augmented generalized Lagrange function is constructed to solve for the optimal solution of each component. The expression of the Lagrange function can be represented as follows:

(5) $$\begin{array}{l} L\left(\{\mu_{k}\},\;\{\omega_{k}\},\;\lambda \right)=\begin{array}{c} \alpha {\sum_{k}\left\|\partial_{t}[\delta (t)+j/\pi t\times u_{k}(t)]e^{-jw_{k}t}\right\|}_{2}^{2}+{\left\|f(t)-\sum_{K=1}^{K}\mu (t)\right\|}_{2}^{2}\\ +\langle \lambda (t),f(t)-\sum_{k}u_{k}(t)\rangle \end{array} \end{array}$$

The computation of the variational problem, as described by the Lagrange function, involves using the alternating direction multiplier method. This method produces different values for the mode components, $\left\{\mu_{k}\right\}$, and the center frequency, $\left\{\omega_{k}\right\}$, during the updating process. The iterative formulas for updating the mode components, $\left\{\mu_{k}\right\}$, and the center frequency, $\left\{\omega_{k}\right\}$, are shown in Equation (6) and Equation (7), respectively. After updating the values of these two parameters, the multiplier value $\hat{\lambda }$ is updated according to Equation (8). Subsequently, Equation (9) is verified to determine if it holds. When Equation (9) is satisfied, update of the multiplier operator, $\hat{\lambda }$, is completed.

(6) $${\hat{\mu }}_{k}^{n+1}\left(\omega \right)=\frac{\hat{f}\left(\omega \right)\sum_{i\neq k}{\hat{\mu }}_{i}\left(\omega \right)+\frac{\hat{\lambda }\left(\omega \right)}{2}}{1+2\alpha {\left(\omega -\omega_{k}\right)}^{2}}$$

(7) $$\omega_{k}^{n+1}=\frac{\int_{0}^{\infty }\omega |{\hat{\mu }}_{k}\left(\omega \right){|}^{2}d\omega }{{\int_{0}^{\infty }\left|{\hat{\mu }}_{k}\left(\omega \right)\right|}^{2}d\omega },k\in \left\{1,2,3\ldots K\right\}$$

(8) $${\hat{\lambda }}^{n+1}\left(\omega \right)={\hat{\lambda }}^{n}\left(\omega \right)+\tau \left(\hat{f}\left(\omega \right)-\sum_{k=1}^{K}{\hat{\mu }}_{k}^{n+1}\left(\omega \right)\right)$$

(9) $$\sum_{k=1}^{K}{\left\|{\hat{\mu }}_{k}^{n+1}-{\hat{\mu }}_{k}^{n}\right\|}_{2}^{2}/\sum_{k=1}^{K}{\left\|\hat{\mu }\right\|}_{2}^{2}<\varepsilon$$

where ${\hat{\mu }}_{k}^{n+1}(\omega )$ denotes the Wiener filtering of the current signal; the real part is $\left\{\mu_{k}\left(t\right)\right\}$ after Fourier transform.

Indeed, finding the optimal parameter combination that matches the signal being analyzed is crucial for the VMD method. The effectiveness and performance of VMD heavily rely on selecting appropriate parameter values for the mode component, K, and penalty factor, α.

### 2.2. K-SVD Basic Principle

The K-SVD algorithm is an iterative process that alternates between sparse coding and dictionary updating. Its core idea is to update the atoms of the dictionary one by one. The objective optimization function of the K-SVD algorithm can be represented as follows:

(10) $$\arg\underset{D,X}{\min}{\left\|Y-DX\right\|}_{F}^{2},s.t.\forall_{i},\left\|x_{j}\right\|\leq T_{0}$$

where Y is the sample matrix formed by the original signal; D is the learning dictionary matrix; X is the sparse coefficients matrix; $x_{j}$ is the *j*th row of X; and $T_{0}$ is the sparsity.

After fixing the dictionary, D, and updating the sparse coefficients, $x_{j}$, using the OMP algorithm, the next step in the K-SVD algorithm is to update the dictionary column by column. This involves calculating the error matrix of the updated atoms, $E_{k}$. The error matrix can be calculated as follows:

(11) $${\left\|Y-DX\right\|}_{F}^{2}={\left\|Y-\sum_{j=1}^{k}d_{j}x_{j}\right\|}_{F}^{2}={\left\|\left(Y-\sum_{j\neq k}d_{j}x_{j}\right)-d_{k}x_{k}\right\|}_{F}^{2}={\left\|E_{k}-d_{k}x_{k}\right\|}_{F}^{2}$$

(12) $$E_{k}=Y-\sum_{j\neq k}a_{j}d_{T}^{j}$$

where $d_{j}$ is the *j*th column of the dictionary, D; $x_{j}$ is the jth row of the sparse matrix, X; $d_{k}$ is the kth column of the dictionary, D; and $x_{k}$ is the kth row of the sparse matrix, X. Determine the set of positions, Ω, of all non-zero entries, $x_{j}$, in the sparse matrix, X, and according to Ω, select the corresponding column of E in $E_{k}$, and perform the singular value decomposition of E, i.e.:

(13) $$E=U\Delta V^{T}$$

The atoms in the dictionary matrix are updated using the first column of the matrix, U. The value of the first column of the matrix, V, multiplied by the corresponding sparse coefficient, $x_{j}$, is used to update the atoms in the dictionary. This process is repeated for each column of the matrix, completing the update of the sparse representation dictionary.

### 2.3. Improved Arithmetic Optimization Algorithm (IAOA)

In the paper by Abualigah [25], the arithmetic optimization algorithm (AOA) is introduced as a new heuristic optimization algorithm based on physical methods. The AOA incorporates the concept of arithmetic operators as an exploration strategy, which enhances the algorithm’s global exploration capability and helps avoid trapping in local optima to some extent. It has been found to be more effective compared with other algorithms such as grey wolf optimization (GWO), particle swarm optimization (PSO), cuckoo search (CS), etc. In this study, AOA was used as the base algorithm, and four arithmetic operators, namely, multiplication (M), division (D), subtraction (S), and addition (A), were employed to further enhance the exploration ability of the algorithm. Similarly to most heuristic optimization algorithms, the AOA operates by generating a set of random solutions (X) and iteratively updating these solutions through the algorithm’s exploration process until an optimal solution is achieved. The stochastic expression for the solution is represented in Equation (14):

(14) $$X=\left[\begin{array}{cccc} x_{1,1} & x_{1,2} & \cdots & x_{1,n}\\ x_{2,1} & x_{2,1} & \cdots & x_{2,n}\\ \vdots & \vdots & \cdots & \vdots \\ x_{N,1} & x_{N,2} & x_{N,3} & x_{N,n} \end{array}\right]$$

where *N* is the number of important parameters in the problem to be solved by the algorithm and *n* is the number of predefined populations.

The exploration capability in the arithmetic optimization algorithm (AOA) is regulated by the mathematical optimizer acceleration (MOA) function. The MOA function is determined by a linear time, t, a predefined minimum value, *MOA*_min_, a predefined maximum value, *MOA*_max_, and the maximum number of iterations, T. The formula for calculating the MOA function is shown in Equation (15):

(15) $$MOA(t)=MOA_{\min}+t\times \left(\frac{MOA_{\max}-MOA_{\min}}{T}\right)$$

In addition to the MOA function, another key parameter in AOA is the mathematical optimizer probability (MOP). The MOP parameter is utilized to balance the exploration and exploitation aspects of the algorithm. It is defined by the equation shown in Equation (16):

(16) $$MOP(t)=1-{\left(\frac{t}{T}\right)}^{\frac{1}{\alpha }}$$

where *α* is the sensitivity parameter.

In the AOA, the exploration behavior differs from other optimization algorithms. The AOA uses the M operator or the D operator to jointly determine its exploration behavior. On the other hand, the S operator and the A operator are employed to generate high-density results. The outcomes obtained from different operators exhibit distinct characteristics, ensuring the effectiveness of the AOA’s exploration capability and exploration behavior. To determine the selection of operators during the exploration process, random values $r_{1}$, $r_{2}$, and $r_{3}$, which range from 0 to 1, are introduced. These random values are used to guide the decision-making process and determine which operator to apply at a particular stage of exploration. By incorporating randomization, the AOA introduces a stochastic element in the selection of operators, enhancing the algorithm’s exploration capability, and promoting diverse exploration paths.

If $MOA<r_{1}$, M and D operators are used in the AOA, the position update rule during this search phase is described by Equation (17).

(17) $$x_{i,j}(t+1)=\{{}_{x_{best}(t)\times MOP\times ((ub_{j}-lb_{j})\times \mu +lb_{j}),\;\;\;\;\;\;\mathrm{else}}^{x_{best}(t)÷(MOP+\varepsilon )\times ((ub_{j}-lb_{j})\times \mu +lb_{j}),\;r_{2}<0.5}$$

where $x_{i,j}(t+1)$ is the calculated new position, $x_{best}(t)$ is the best position obtained so far, ε is the epsilon number used to avoid division by zero, $lb_{j}$ and $ub_{j}$ denote the lower and upper bounds of the *j*th dimension of the variable, respectively, and μ is a parameter empirically set to 0.5.

If $MOA>r_{1}$ is used, it is calculated using the S operator and the A operator. When $r_{3}<0.5$, the S operator is used; vice versa, the A operator is used. The specific position update rule during this search phase is represented by Equation (18):

(18) $$x_{i,j}(t+1)=\{{}_{x_{best}(t)+MOP\times ((ub_{j}-lb_{j})\times \mu +lb_{j}),\;\;else}^{x_{best}(t)-MOP\times ((ub_{j}-lb_{j})\times \mu +lb_{j}),\;r_{3}<0.5}$$

The initialization of the population is a critical step in the AOA that can significantly impact the algorithm’s performance. Random distribution in the initialization process can lead to an uneven distribution of individuals in the initial population, which may increase the risk of the algorithm becoming trapped in local optima and limit its global search ability. To address this issue, chaotic variables are introduced in the population initialization phase. Chaotic variables exhibit characteristics such as randomness, ergodicity, and regularity, which can enhance the exploration capability of the algorithm [26]. In this study, the combination of three types of chaotic mapping functions, namely, logistic, sine, and cosine, was employed. This combination, known as logistic–sine–cosine (LSC) mapping, offers more complex chaotic performance [27]. By utilizing LSC mapping to initialize the population in the AOA, the algorithm benefits from the enhanced exploration capabilities provided by chaotic variables. This approach aims to improve the algorithm’s ability to escape local optima and enhance its global search ability, ultimately leading to better optimization results.

The expression for logistic mapping is described by Equation (19):

(19) $$x_{i+1}=4rx_{i}(1-x_{i})\begin{array}{cc} & r\in [0,1] \end{array}$$

The expression for sine mapping is described by Equation (20):

(20) $$x_{i+1}=r\sin(\pi x_{i})\begin{array}{cc} & r\in [0,1] \end{array}$$

The expression for tent mapping is described by Equation (21):

(21) $$x_{i+1}=\{\begin{array}{cc} 2rx_{i} & \\ 2r(1-x_{i}) & \end{array}\begin{array}{c} x_{i}<0.5\\ x_{i}\geq 0.5 \end{array}$$

The expression for logistic–sine–cosine mapping is described by Equation (22):

(22) $$x_{i+1}=\begin{array}{c} \cos(\pi (4r_{4}x_{i}(1-x_{i})\\ +(1-r_{4})\sin(\pi x_{i})-0.5)) \end{array}\;r_{4}\in [0,1]$$

In the context of opposition-based learning (OBL), a learning strategy that directly affects population initialization, there is a technique known as the inverse approximation of the number of opposites. This technique aims to address the issue of uneven distribution of population positions more effectively while reducing computational complexity.

We define a hypothesis, x∈[a,b], whose inverse proximal opposites, $x_{qr}$, are defined as follows:

(23) $$x_{qr}=rand(c,x)$$

where c=(a+b)/2 is the center of [a,b] [28].

To expedite the convergence towards the optimal solution and achieve a more uniform distribution of solution seekers in the population initialization of the AOA, a multi-step process is employed. Initially, a set of initial values is generated using logistic–sine–cosine (LSC) chaotic mapping. Then, contrastive learning is applied to generate a new set of samples using the initial values as references. These samples are substituted into a specific function, and those with the minimum fitness value are continuously recorded. This process yields a set of positions that are closer to the optimal solution compared with the previous methods. These positions are encoded into variable X and utilized as the initial solution seekers for subsequent computations in the algorithm.

The exploration behavior of different optimization algorithms can vary, leading to different optimization processes for various functions. To further enhance the optimization capability of AOA, an adaptive Gaussian distribution is introduced to improve its exploration process [29]. When the global optimal solution of the AOA remains unchanged for a prolonged period, indicating that further computation and improvement are unlikely to yield better results, Gaussian distribution is employed to generate a new solution. This helps to introduce diversity and exploration into the search process. The specific equation depicting the usage of Gaussian distribution for creating a new solution is presented in Equation (24).

(24) $$x_{inew}=\left\{\begin{array}{cc} Gaussian(x_{i,j},\mu ,\sigma )+(x_{i,j}-r_{5}\times x_{i}), & r_{6}<0.5\\ Gaussian(x_{i,j},\mu ,\sigma ), & \mathrm{else} \end{array}\right\}$$

where $r_{5}$ and $r_{6}$ are the random values in [0, 1], $Gaussian(x_{i,j},\mu ,\sigma )$ is the Gaussian distribution, $x_{i}$ is the current position, μ is the mean, and σ is the standard deviation. In order to improve the exploration ability of the algorithm, the Gaussian distribution is no longer calculated with the global optimal position as the sample, but with all the global positions as the reference and redistributed, so as to obtain a new set of global variables again, which, in turn, improves the exploration ability of the algorithm.

**Figure 1:**
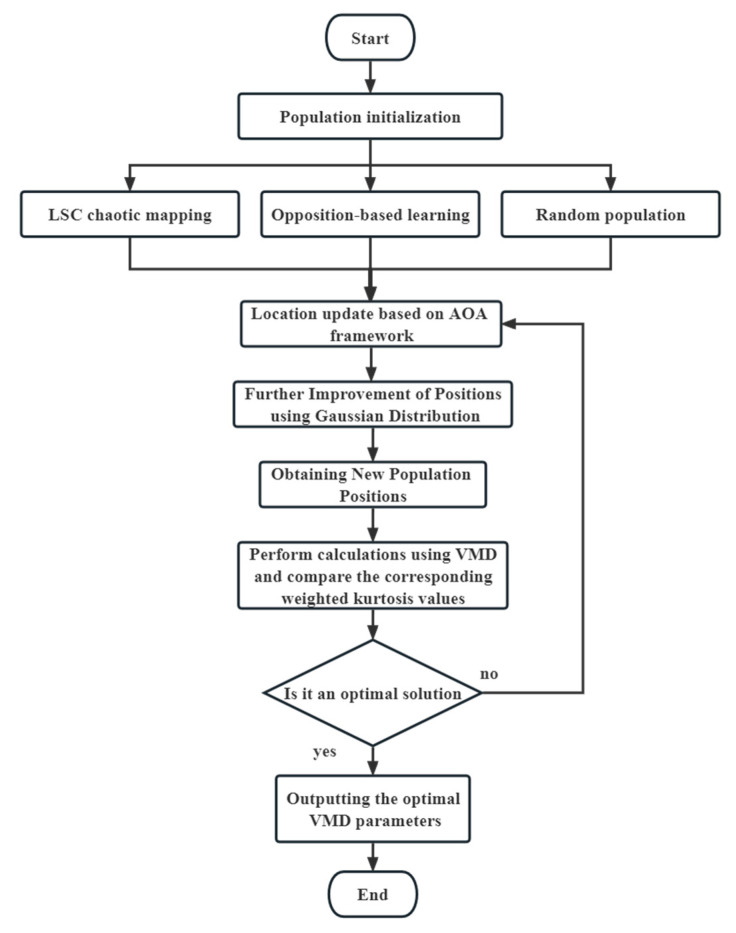
The IAOA optimization flowchart.

The flow of the improved artificial organism algorithm (IAOA) is illustrated in Figure 1.

### 2.4. Flow Chart for the Signal Enhancement of Rolling Bearing Fault Characteristics

In this study, a fault feature signal enhancement process is proposed, which involves several key steps. Firstly, the acquired raw signals are subjected to decomposition using VMD. To optimize the VMD parameters, specifically the mode component, K, and the penalty factor, α, the IAOA is employed. The IAOA ensures effective parameter optimization for VMD.

The resulting IMFs obtained from the VMD serve as the original dictionary, capturing the underlying fault features present in the signal. Subsequently, the K-SVD algorithm, a widely used dictionary learning technique, is applied for noise reduction on the original dictionary. Through iterative updates of the dictionary and sparse coefficients, K-SVD achieves noise reduction and signal enhancement.

The noise-reduced dictionary, obtained from the K-SVD process, is then utilized to reconstruct the signal by multiplying it with the sparse coefficient matrix. This multiplication effectively combines the dictionary elements, weighted by the sparse coefficients, resulting in the reconstruction of a noise-reduced and enhanced signal.

**Figure 2:**
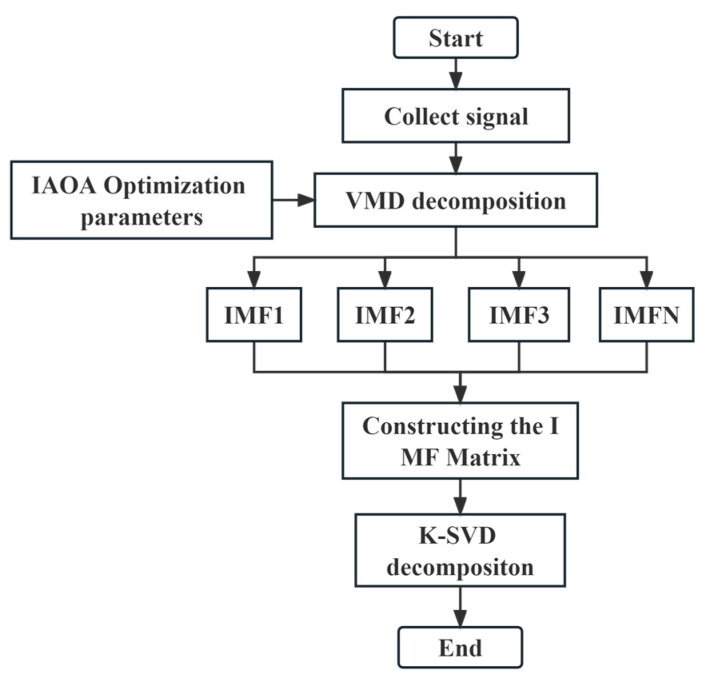
Flowchart depicting the fault characteristic signal enhancement process.

Overall, the proposed fault feature signal enhancement process, as depicted in Figure 2, involves the decomposition of raw signals using VMD with parameter optimization through IAOA. The resulting IMFs form the original dictionary, which undergoes noise reduction using K-SVD. The reconstructed signal, obtained through multiplication of the noise-reduced dictionary with the sparse coefficients, represents an enhanced version of the fault feature signal.

## 3. Experimentation and Analysis

### 3.1. Based on Case Western Reserve University Data Analysis

The proposed method in this paper was validated using experimental vibration signal data from the Bearing Signal Library Center of Case Western Reserve University (CWRU) [30]. The data pertained to the SKF 6205-2RSJEM SKF bearing, with a motor speed of 1797 r/min. The analyzed signal represented the inner ring failure signal, with a loss diameter of 0.1778 mm. The vibration signals were sampled at a frequency of 12 kHz for a duration of 1s. The inner ring failure frequency, $f_{i}$, was determined to be 162 Hz, while the bearing rotation frequency, $f_{o}$, was measured at 30 Hz.

**Figure 3:**
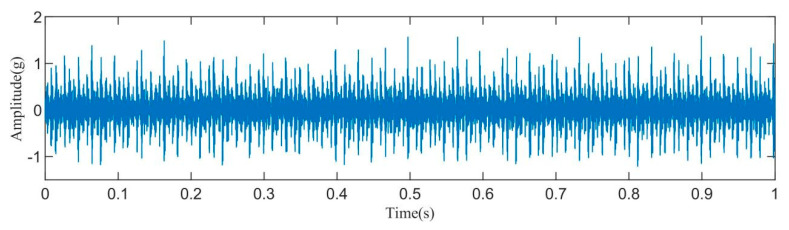
Time–domain diagram of the original fault signal.

**Figure 4:**
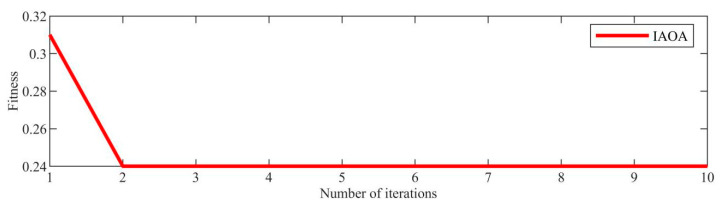
Iteration of the fitness function.

Figure 3 displays the time–domain representation of the fault signal. Due to a significant amount of noise present in the original signal, an initial step involved decomposing the original signal using the IAOA–VMD method. The iterative convergence of the fitness function for the IAOA–VMD algorithm is depicted in Figure 4. It is evident from the graph that the IAOA algorithm, enhanced by LSC mapping and adversarial learning, achieves the optimal value during the second iteration. The optimized VMD parameters consist of a mode component, K, value 12, and a penalty factor, α, value 7250.

**Figure 5:**
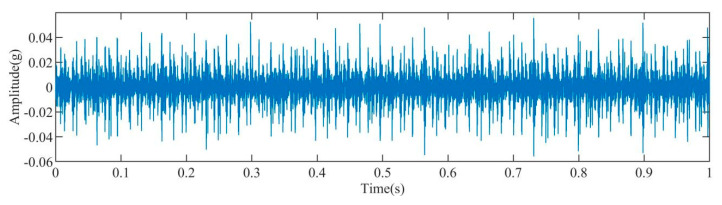
Enhanced signal time–domain diagram.

Under the premise of guaranteeing parameter optimization, the VMD is decomposed to obtain 12 IMF components. The feature matrix is constructed with this IMF component to obtain a 12 × 12,000 feature matrix. The obtained feature matrix is used as the original dictionary for K-SVD. By multiplying the obtained dictionary and sparse coefficient matrix to obtain the signal components after noise reduction, the method is used to realize the noise reduction and enhancement of the bearing fault feature signal. The time–domain diagram of the enhanced signal is shown in Figure 5, from which it can be seen that the noise has been significantly reduced.

**Figure 6:**
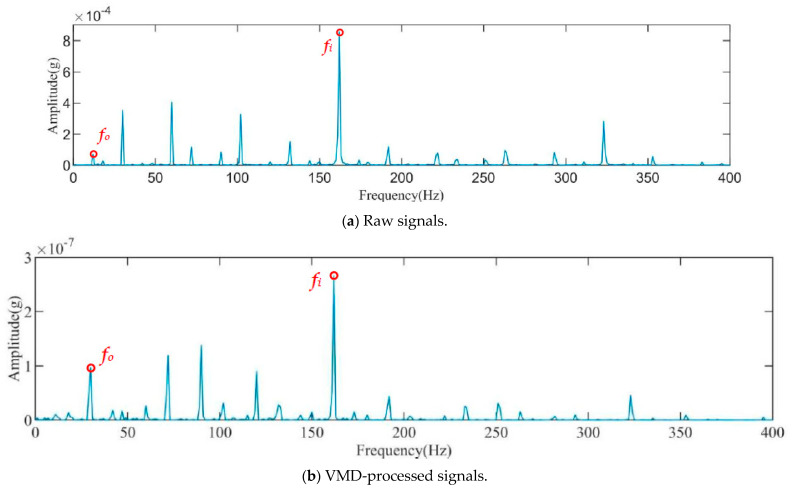

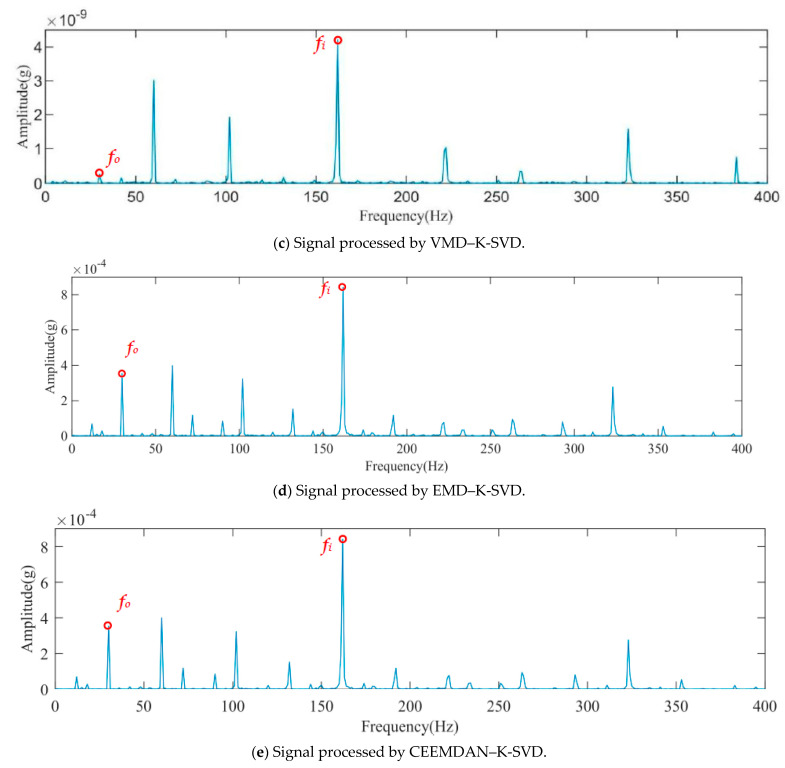
Squared-envelope spectrum analysis.

To assess the effectiveness of noise reduction and signal enhancement, the squared-envelope spectrum analysis of the faulty signal processed by VMD–K-SVD is performed and compared with the original signal and the signal processed by VMD. The results are depicted in Figure 6a–c. Additionally, to evaluate the impact of different signal decomposition methods combined with K-SVD, the EMD and CEEMDAN algorithm are employed in conjunction with the K-SVD algorithm to perform noise reduction of the vibration signals. The squared-envelope spectrum analysis is then applied; the outcomes are presented in Figure 6d,e. Figure 6a illustrates that, aside from the fault eigenfrequency, the signal contains a significant amount of irrelevant noise that can potentially interfere with fault diagnosis outcomes. In Figure 6b, the squared-envelope spectrum following VMD processing exhibits more prominent bearing rotation frequency, rotation multiplier frequency, and inner ring fault frequency when compared with the original signal. However, the amplitude of the inner ring fault remains less conspicuous in comparison to the rotation frequency. Figure 6c demonstrates that, in contrast to the original signal and the squared-envelope spectrum after VMD processing, the squared-envelope spectrum subsequent to VMD–K-SVD processing exhibits more pronounced bearing rotation frequency, multiplicative frequency, and fault frequency. A comparison of the amplitude of the processed fault characteristic frequency and the rotation frequency allows for the determination of the bearing’s operational status and fault type.

### 3.2. Signal Analysis of Rolling Bearings in a Thrust Bearing Test Rig

We considered the limitations of the bearing dataset available in Case Western Reserve University (CWRU) and, in order to demonstrate the applicability of the proposed method to signals with high sampling frequency and high noise, this study focused on acoustic signals generated during the operation of a thrust bearing test bench. The VMD–K-SVD method was employed to perform noise reduction and enhancement on these signals.

**Figure 7:**
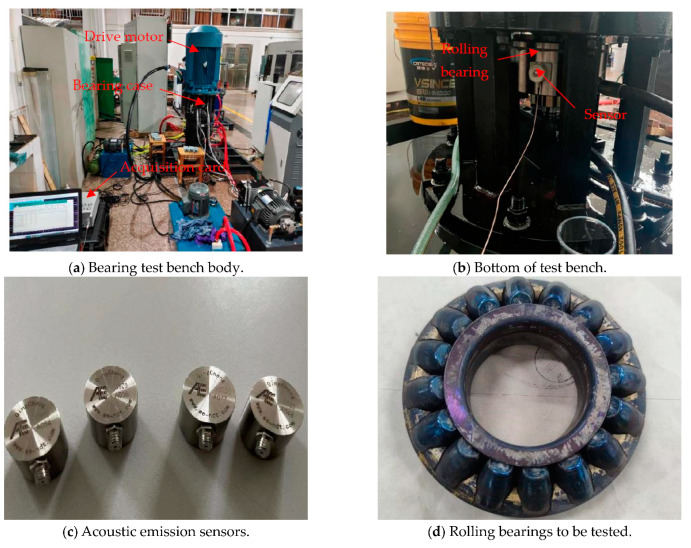
Experimental setup.

The thrust bearing test rig, depicted in Figure 7a, was primarily designed to measure changes in oil film pressure and oil film temperature of large plain bearings during operation. The test rig incorporated 29412E type roller bearings in its bearing box. Acoustic signals from this rolling bearing were collected by placing an acoustic signal sensor on the outer side of the box, as illustrated in Figure 7b. The specific model and style of the sensor are presented in Figure 7c. During the experiment, the motor speed was set to 2970 r/min, and the acoustic signals were sampled at a frequency of 1000 kHz for a duration of 1s. This resulted in a dataset containing 1,000,000 data points, as shown in Figure 7d. To ensure the experiment’s authenticity, a naturally damaged rolling bearing was used, reflecting wear and tear accumulated during long-term usage. Table 1 provides the relevant parameters associated with the rolling bearing. Based on these parameters, the rotation frequency, $f_{o}$, and the outer ring failure frequency, $f_{i}$, of the rolling bearing were calculated to be 49.5 Hz and 323 Hz, respectively.

**Figure 8:**
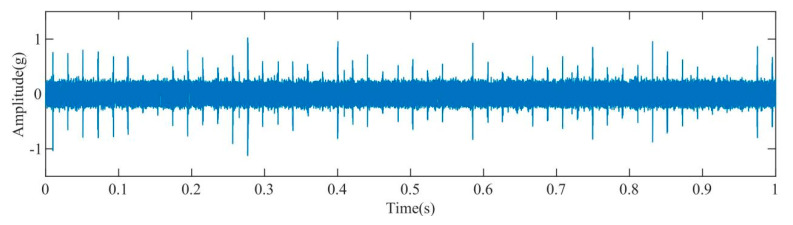
Time–domain diagram of the original signal.

Figure 8 presents the time–domain diagram of the acquired acoustic signals from the rolling bearing. Comparison between this diagram and the time–domain diagram of the vibration signals shown in Figure 3 reveals that the acoustic signals in Figure 8 contain a significant amount of impact noise and periodic noise. The presence of such noise components can significantly impact the accuracy and reliability of subsequent analysis and fault diagnosis processes.

**Figure 9:**
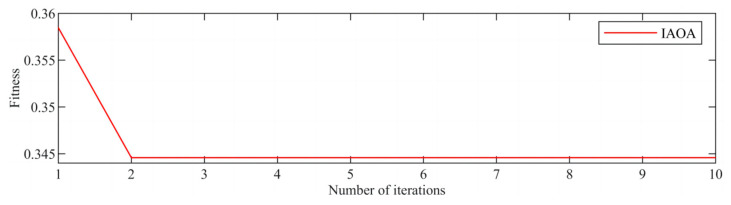
VMD iteration diagram.

To reduce noise and enhance the fault signal in the acoustic signal of the rolling bearing, an adaptive decomposition and reduction process was performed using IAOA–VMD. The iterative diagram of the fitness function for the IAOA–VMD algorithm is depicted in Figure 9. It can be observed that the IAOA algorithm achieved the optimal value during the second iteration. The optimized parameters obtained from the IAOA–VMD optimization search, namely, the mode component, K (12), and the penalty factor, α (9910), were then used in the VMD of the original acoustic signal. This decomposition step aims to effectively separate the signal components for further analysis and processing.

After substituting the optimal parameters into the VMD, 12 IMFs components were obtained. These IMF components were then used to construct a feature matrix with dimensions of 12 × 12,000. This feature matrix served as the original dictionary for the subsequent K-SVD.

**Figure 10:**
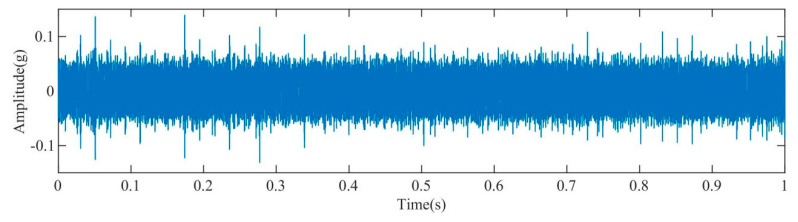
Enhanced signal time–domain diagram.

By applying the K-SVD using the constructed feature matrix, the signal after enhancing the fault signal was obtained. Figure 10 illustrates the time–domain diagram of the enhanced signal. Comparing it with the original signal, it is evident that the enhanced signal successfully removed a substantial amount of shock noise while significantly improving the smoothness of the periodic signal.

To further investigate the amplitude relationship between the corresponding fault frequency and the rotation frequency in the enhanced signal, squared-envelope analysis was performed on the original signal, the VMD-processed signal, and the VMD–K-SVD-processed signal.

**Figure 11:**
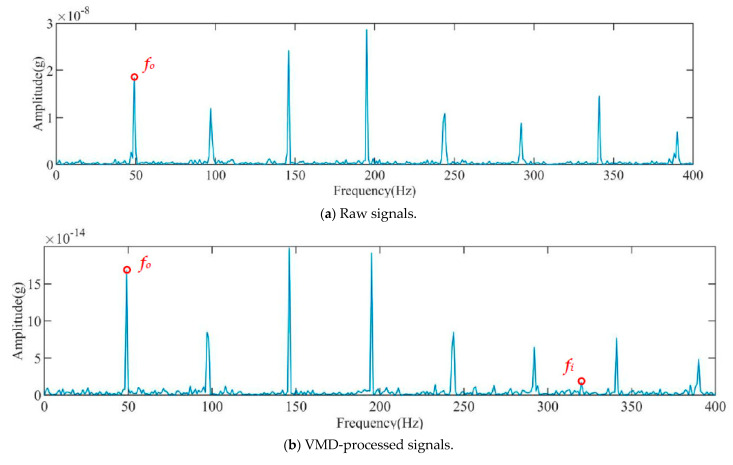

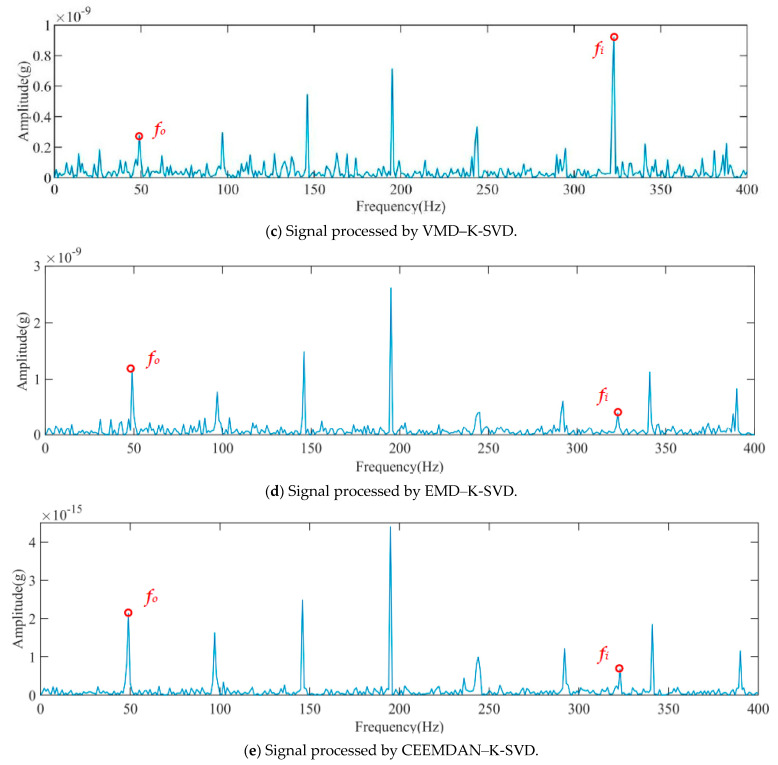
Squared-envelope spectrum analysis.

Figure 11a–c display the squared-envelope spectra of the original signal, the VMD-processed signal, and the VMD–K-SVD-processed signal, respectively.

From Figure 11a, only the rotation frequency of the rolling bearing and its octave frequency are visible, while the fault frequency of the bearing cannot be distinguished clearly. Figure 11b demonstrates that the amplitude of the fault signal was significantly improved compared with the original signal. However, the fault frequency is still not clearly discernible. In contrast, Figure 11c shows that the amplitude of the fault characteristic frequency is significantly enhanced compared with the previous spectra. It is now possible to clearly distinguish the rotation frequency of the rolling bearing and accurately determine its operational status. This result confirms the effectiveness of the proposed method for enhancing weak fault signals.

The squared-envelope spectrum of the original signal, VMD-processed signal, VMD–K-SVD-processed signal, EMD–K-SVD-processed signal, and CEEMDAN–K-SVD-processed signal are presented in Figure 11a, b, c, d, and e, respectively.

In Figure 11a, only the rotation frequency of the rolling bearing and its octave frequency are visible, while the fault frequency of the bearing cannot be distinguished. Figure 11b demonstrates a noticeable improvement in the amplitude of the fault signal; however, it is still not clearly evident. Conversely, Figure 11c shows a significant improvement in the amplitude of the fault characteristic frequency compared with the previous cases. The rotation frequency of the rolling bearing is clearly distinguishable, enabling the determination of its operational status. This substantiates the effectiveness of the proposed method for weak fault signals. Based on the comparison of Figure 11c–e, it can be observed that the amplitude of the fault frequency of the rolling bearing in the signals processed by EMD–K-SVD and CEEMDAN–K-SVD is enhanced compared with that of VMD. However, the effect is still weaker than that achieved by the VMD–K-SVD method proposed in this paper.

## 4. Conclusions

This paper addresses the challenge of separating noise in rolling bearing signals by proposing an effective method that combines VMD with K-SVD for the signal enhancement of bearing fault characteristics.

(1) The optimized VMD algorithm based on the IAOA is capable of effectively separating the overall noise into individual IMF components, thereby improving the clarity of each IMF.
(2) By subjecting the original dictionary, constructed using the IMF components, to K-SVD processing, the embedded noise is significantly reduced. This noise reduction step contributes to the enhancement of the fault characteristic signals, resulting in improved signal quality.
(3) The effectiveness of the proposed method is demonstrated through two sets of experiments involving low-sampling-frequency vibration signals and high-sampling-frequency acoustic signals. These experiments validate the method’s ability to reduce noise and enhance fault characteristic signals, consequently enhancing the accuracy of fault diagnosis. However, it is important to note that the datasets used in this paper are experimental data and may not fully represent the high noise conditions encountered in complex machinery. Therefore, further testing and validation under real working conditions with highly noisy data are necessary to confirm the method’s effectiveness.

It is crucial to conduct additional research and experimentation to evaluate the proposed method’s performance in real-world scenarios with complex machinery and high-noise environments.

## Figures and Tables

**Table 1:** Parameters related to rolling bearings to be tested.

Parameters	Bearing Type	Inner Ring Diameter/mm	Outer Ring Diameter/mm	Thickness/mm	Bearing Contact Angle/°	Pitch Diameter/mm	Number of Scrollers
Numerical value	29412E	60	130	42	30	95	16

## Data Availability

The CWRU data can be found at https://engineering.case.edu/bearingdatacenter/12k-drive-end-bearing-fault-data (accessed on 20 December 2021).
